# 228. Real-world Abrysvo Vaccine Effectiveness (VE) against Respiratory Syncytial Virus (RSV)-related Lower Respiratory Tract Disease (LRTD) Hospitalizations and Emergency Department Admissions over Two RSV Seasons—Kaiser Permanente of Southern California (KPSC), October 2023–April 2025

**DOI:** 10.1093/ofid/ofaf695.011

**Published:** 2026-01-11

**Authors:** Sara Y Tartof, Negar Aliabadi, Gabriella Goodwin, Jeff Slezak, Vennis Hong, Bradley Ackerson, Qing Liu, Sally Shaw, Sabrina Welsh, Banshri Kapadia, Brigitte Spence, Gregg S Davis, Joseph Lewnard, Hina Chowdhry, Timothy B Frankland, Michael Aragones, Michael p Dutro, Erica Chilson, Evan Zasowski, Luis Jodar, Bradford D Gessner, Elizabeth Begier

**Affiliations:** Kaiser Permanente Southern California, Pasedena, CA; Pfizer, New York, NY; Kaiser Permanente, Southern California, Pasadena, California; Kaiser Permanente Southern California, Pasedena, CA; Kaiser Permanente Southern California, Pasedena, CA; Kaiser Permanente Southern California, Pasedena, CA; Pfizer Inc., Collegeville, Pennsylvania; Kaiser Permanente Southern California, Pasedena, CA; Pfizer, Inc, Collegeville, Pennsylvania; Kaiser Permanente Southern California, Pasedena, CA; Kaiser Permanente Southern California, Pasedena, CA; Kaiser Permanente Southern California, Pasedena, CA; University of California, Berkeley, Berkeley, California; Kaiser Permanente, Fountain Valley, California; KP Center for Integrated Health Care Research (CIHR), Honolulu, Hawaii; Kaiser Permanente Southern California, Pasedena, CA; Pfizer, Inc, Collegeville, Pennsylvania; Pfizer, New York, NY; Pfizer, New York, NY; Pfizer, New York, NY; EpiVac Consulting, Anchorage, Alaska; Pfizer Vaccines, Dublin, Dublin, Ireland

## Abstract

**Background:**

Abrysvo was approved for use in older adults in 2023. We evaluated Abrysvo VE against RSV-related LRTD in KPSC, a large integrated healthcare system, to estimate long-term protection.

**Table 1.**
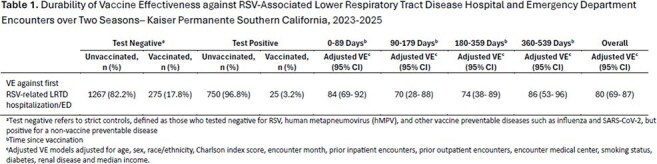
Durability of Vaccine Effectiveness against RSV-Associated Lower Respiratory Tract Disease Hospital and Emergency Department Encounters over Two Seasons─ Kaiser Permanente Southern California, 2023-2025

**Table 2.**
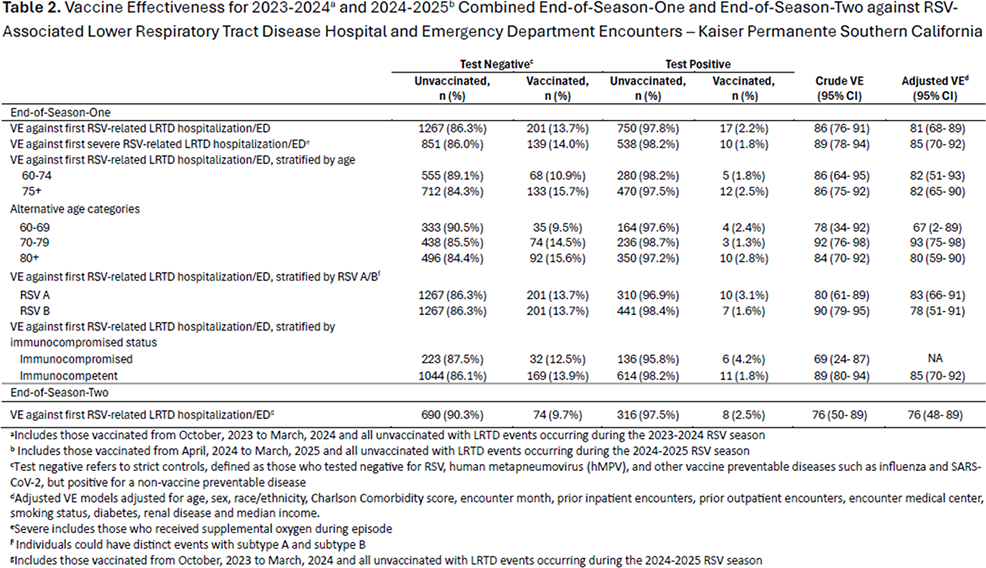
Vaccine Effectiveness for 2023-2024a and 2024-2025b Combined End-of-Season-One and End-of-Season-Two against RSV-Associated Lower Respiratory Tract Disease Hospital and Emergency Department Encounters ─ Kaiser Permanente Southern California

**Methods:**

We assessed Abrysvo VE among adults aged ≥ 60 years through electronic health records plus enhanced specimen testing using a test-negative case-control study over 2 seasons (10/2/2023 – 4/17/2025). The analysis included hospitalization or emergency department (ED) visits with LRTD ICD-10 discharge codes and respiratory specimen tested on cobas eplex RP2. Cases were RSV+. Controls were negative for RSV, hMPV, flu, SARS-CoV-2, and positive for a non-vaccine preventable disease. Exposure was Abrysvo receipt ≥ 21 days before LRTD. Odds ratios (OR) and 95% CI were estimated from multivariable logistic regression (VE=1−OR X 100%).

**Results:**

Overall, 2,317 events contributed to the 2-season VE waning analysis, 775 (33.4%) were RSV+; 25 (3.2%) were vaccinated, compared with 275 (17.8%) vaccinated events among 1,542 controls (Table 1). Median time since vaccination (TSV) was 113 days (IQR 63-313). VE against RSV-related LRTD hospitalization/ED was 80% (69–87) across 2 seasons overall, with consistent results in the most distal period 360–539 days from vaccination : 86% (53–96). In the analysis limited to patients in their 2nd season after vaccination (n=1,088 events), median TSV was 373.5 days (IQR 343-403) and VE was 76% (48–89) (Table 2).

Among 2,235 events contributing to the EOS1 analysis, 767 (34.3%) were RSV+; 17 (2.2%) were vaccinated compared with 201 (13.7%) vaccinated among 1,468 controls. Median TSV was 84.5 days (IQR 53-126). Median (IQR) age was 77 years (70–85), 72% had Charlson Comorbidity score ≥ 3, and 17.8% were immunocompromised. EOS1 VE was 81% (95% CI:68–89), remaining consistent for persons ≥ 80 years (80% [59–90]) and with severe LRTD (85% [70–92]). VE for RSV A was 83% (66–91) and for RSV B 78% (51–91).

**Conclusion:**

Abrysvo was highly effective against RSV-associated LRTD hospitalization/ED visits without evidence of VE waning during its first 2 seasons of use. VE was similarly high among older age groups, those with severe disease, and by RSV subtype. These results expand on prior data by evaluating endpoints through two seasons and by RSV subtype.

**Disclosures:**

All Authors: No reported disclosures

